# Changes in Composition and Diversity of Epiphytic Microorganisms on Field Pea Seeds, Partial Crop Peas, and Whole Crop Peas during Maturation and Ensiling with or without Lactic Acid Bacteria Inoculant

**DOI:** 10.1128/spectrum.00953-22

**Published:** 2022-08-10

**Authors:** Martin Bachmann, Monika Wensch-Dorendorf, Christian Kuhnitzsch, Sabine Kleinsteuber, Denny Popp, Annabel Thierbach, Siriwan D. Martens, Olaf Steinhöfel, Annette Zeyner

**Affiliations:** a Martin Luther University Halle-Wittenberg, Institute of Agricultural and Nutritional Sciences, Halle (Saale), Germany; b Saxon State Office for Environment, Agriculture and Geology, Köllitsch, Germany; c Helmholtz Centre for Environmental Research (UFZ), Department of Environmental Microbiology, Leipzig, Germany; Yeungnam University

**Keywords:** field pea, maturity, silage quality, epiphytic bacteria, eukaryotes

## Abstract

The present study was conducted under the hypothesis that, in field peas, type of plant material, stage of maturity, ensiling, silage additive, and aerobic stress affect the composition and diversity of epiphytic microbial communities. Epiphytic microbial composition and diversity of pea seeds, partial crop peas, and whole crop peas was analyzed at different stages of late maturity, before and after ensiling, and with or without the use of lactic acid bacteria (LAB) as inoculant. Suitable combinations among pea crop variants, maturity stages, and inoculant use for the production of stable silages with sufficient aerobic stability after opening and during feed-out were identified. Genomic DNA was extracted, and 16S and 18S rRNA gene amplicons were sequenced. To assess the quality of the various silages, nutrient concentration, pH value, concentration of lactic acid, short chain fatty acids, and alcohols, and aerobic stability were determined. Pea seeds were barely colonized by epiphytic microorganisms. In partial and whole crop peas, composition and α-diversity (Shannon index) of bacterial communities did not differ between crop variants but differed among maturity stages. Epiphytic eukaryotes were rarely found on partial and whole crop peas. Bacterial composition and α-diversity were affected by ensiling and subsequent aerobic storage. In partial and whole crop peas, plant maturation caused an increase of the relative abundance of naturally occurring LAB (*Weissella*, *Pediococcus*, and *Lactobacillus* spp.). As a possible result, natural LAB support stable ensiling conditions even without the use of inoculants beginning with a maturity of 78 on the BBCH scale. This corresponded with a dry matter (DM) concentration of 341 and 363 g/kg in partial and whole crop peas, respectively. Addition of LAB inoculants, however, reduced ammonia, acetic acid, and butanol concentrations, and supported aerobic stability. Earlier stages of plant maturity (BBCH 76 and 77, 300 g DM/kg or less) were more prone to microbial spoilage. Stable pea seed silages can be produced at a maturity between BBCH 78 (427 g DM/kg) and 79 (549 g DM/kg), but they undoubtedly require LAB inoculation or application of other ensiling agents.

**IMPORTANCE** Field peas are important protein suppliers for human and animal nutrition. They can be grown in many areas of the world, which may reduce imports of protein plants and has beneficial economic and ecological effects. Ensiling is a method of preserving feed that can be implemented easily and cost-effectively at the farm. Peas harvested as seeds, partial crop, or whole crop at different maturities enable a wide range of applications. The study characterized epiphytic microbial communities on peas in terms of composition and diversity depending on the maturity of the plants and feed conservation by ensiling as they play an essential role for the production of silages. Even if this study did not consider year, site, or cultivar effects, the results would show which part of the plant is probably well suited for the production of stable and high-quality silages and at which stage of maturity.

## INTRODUCTION

Dry pulses such as field peas (Pisum sativum) have gained importance as protein suppliers for human and animal nutrition, although production levels have varied over the years (1–3). In 2018, the worldwide production of dry peas reached 13.5 Mt (7.9 Mha), whereas in Europe, 5.3 Mt of peas were produced on 2.8 Mha (3). They can be grown locally in many areas and may reduce imports of other protein plants such as soybeans. This has both economic and ecological benefits (2).

Fermentation by lactic acid bacteria (LAB) is a traditional and commonly used method of preservation of foods (4) and animal feeds (5). A major challenge in silage making is the control of rapid respiration of carbohydrates and proteolysis by plant enzymes after cutting. Then, microorganisms change the chemical composition of the raw plant material (4) and that way determine silage quality with beneficial or detrimental outcomes (6–13). Silage quality can be controlled by wilting, sufficient pH reduction (if necessary, using inoculants and/or sugar), and provision of continuous anoxic conditions (to avoid growth of proteolytic bacteria, yeasts, and molds before opening of the silage) (5, 10).

Advanced analyses of the composition and diversity of epiphytic microbial communities and the community dynamics during ensiling can provide a better understanding of the ensiling process (14), which is prospectively helpful to adapt control strategies. Such information is currently not available for field peas. Therefore, the current study was conducted to describe the composition and diversity of epiphytic bacteria and eukaryotes on native and ensiled pea seeds, partial crop peas, and whole crop peas at different stages of plant maturity. The silages were made with and without a LAB preparation as inoculant, and were analyzed before and after being exposed to aerobic stress. The hypothesis was that type of plant material, stage of maturity, ensiling, silage additive, and aerobic stress affect the composition and diversity of epiphytic microbial communities.

## RESULTS AND DISCUSSION

### Changes in epiphytic microbial composition throughout pea maturation.

A total of 109 samples with minimal 11 and maximal 193 amplicon sequence variants (ASVs; Table S1 in the supplemental material) and 110 samples with minimal 1 and maximal 75 ASVs (Table S2) were analyzed for the composition of bacterial and eukaryotic communities, respectively. At least 355 and at most 437,064 16S rRNA reads per sample were available (Table S3), whereas a minimum of 38 and a maximum of 257,309 18S rRNA reads per sample were obtained (Table S4). The rarefaction curves are shown in Fig. S1.

Before removal of ASVs assigned to archaea, chloroplasts, or mitochondria, 63,181 to 121,740 16S rRNA reads per sample were available in native pea seeds. The number of 16S rRNA reads per sample that remained was 355 to 59,470, and it was especially low at premature stages (355 to 12,543; BBCH 76 to 78). A number of 63,221 to 164,526 18S rRNA reads per sample was available, but only 38 to 447 18S rRNA reads per sample remained after discarding ASVs assigned to the pea plant (i.e., to the phylum *Charophyta*). This clearly shows that native pea seeds were hardly colonized by bacteria (at least at premature stages) and eukaryotic microorganisms, which can be explained by their protected location in the pod. In native partial and whole crop peas, colonization with epiphytic eukaryotes was, in many samples, low as well. A significant number of 18S rRNA reads per sample remained in partial and whole crop peas only at maturity stages with BBCH 78 and 79 (3,059 to 98,331 reads per sample) (Table S4).

Commensal epiphytes are defined as nonpathogenic microbes (at least for the plant itself) that strictly colonize the plant surface without penetrating plant tissue throughout their life cycles (15). They strike up symbiotic relationships with the plants and other pro- and eukaryotes (16). They support the host plant nutritionally, promote growth, and defend it against biotic and abiotic stressors (16–18). Epiphytic bacterial communities are also antagonists of several pests and pathogens (18–23).

**FIG 1:**
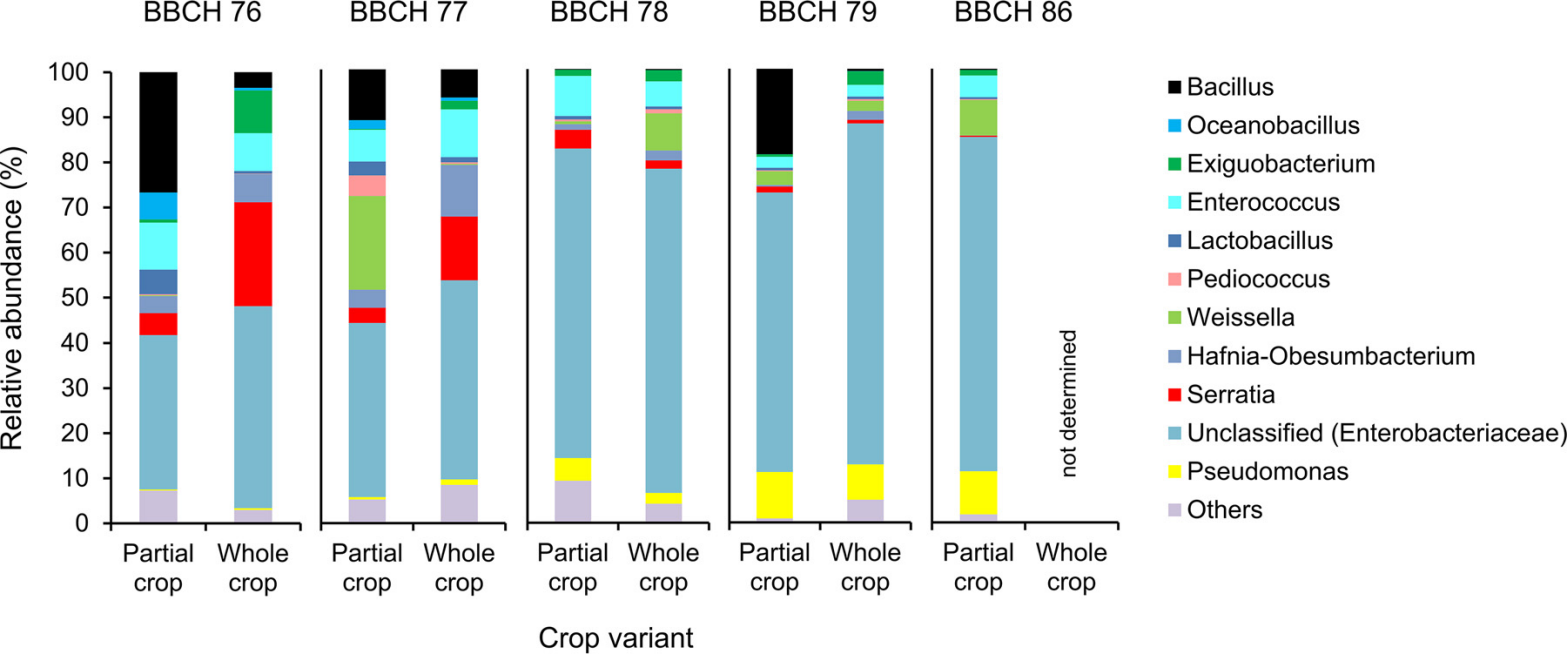
Relative abundances of bacterial genera as percent of total bacteria in native partial and whole crop peas at five stages of maturity. BBCH stages were assigned according to Meier (45) and are specified in Table 2. *Enterobacteriaceae* could not be classified further in every case. Bacteria with a relative abundance lower than 5% are summarized under the term “others.” The number of biological replicates and standard deviations of the means are included in Table S6.

Relative abundance of epiphytic bacteria on native partial and whole crop peas was calculated on the basis of ASVs given in Table S5. The relative abundances are illustrated in Fig. 1 and specified in Table S6. The most abundant epiphytic bacteria belonged to the phyla *Firmicutes* and *Proteobacteria*. Next to unclassified representatives of the family *Enterobacteriaceae*, we found *Bacillus*, *Enterococcus*, Pseudomonas, *Serratia*, and *Weissella* as dominant genera (Fig. 1).

**FIG 2:**
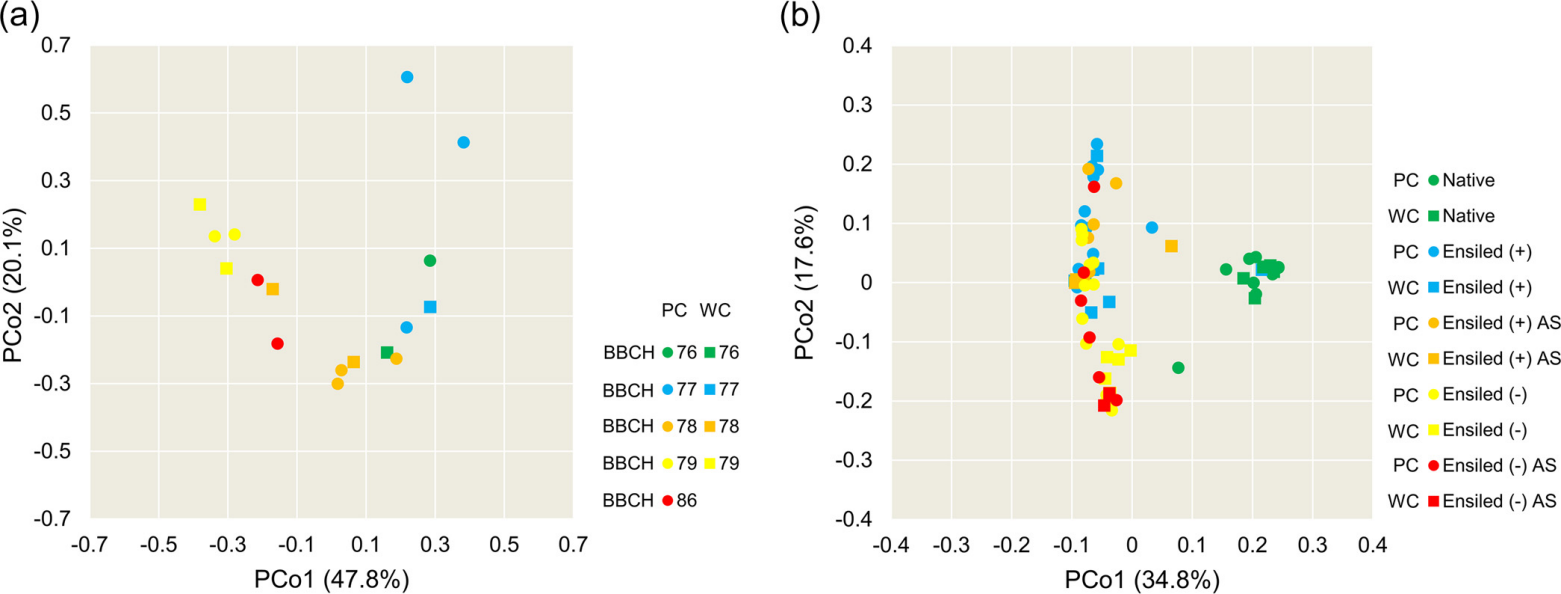
Principal coordinate analysis (PCoA) based on Bray-Curtis similarity of the epiphytic bacterial composition of native field peas (a) discerned according to crop variant and stage of maturity and of the bacterial composition of native and ensiled field peas (b) discerned according to treatment and crop variant. BBCH maturity stages were assigned according to Meier (45) and are specified in Table 2. + denotes addition of microbial inoculant; – denotes ensiling without addition of inoculant; AS, aerobic storage (i.e., silages were stored 7 days under aerobic conditions), PC, partial crop peas, WC, whole crop peas.

The epiphytic bacterial composition did not differ between partial and whole crop peas (*P = *0.208) but differed among the stages of pea maturity (*P < *0.001) (Fig. 2). Premature stages (BBCH 76 to 78) and matured stages (BBCH 79 and 86) were clearly differentiated (Fig. 2). Relative abundances of *Bacillus*, *Enterococcus*, and *Serratia* spp. decreased during maturation of the host plant, whereas those of unclassified *Enterobacteriaceae* and Pseudomonas spp. increased (Fig. 1).

Most of the 18S rRNA reads were assigned to *Opisthokonta*. Next to some insects, the eukaryotic community on partial and whole crop peas mainly comprised unclassified representatives of the *Sporidiobolaceae* family, *Cladosporium* spp., *Pichia* spp., as well as other unclassified fungi.

### Effect of ensiling on microbial composition.

The analysis of native and ensiled peas in one data set revealed that epiphytic bacterial communities differed between crop variants (*P < *0.05) and treatments (*P < *0.001). The PCoA clearly shows that ensiling changed the composition of the bacterial community, whereas aerobic storage of the silages apparently had little effect (Fig. 2). In the silages made without inoculant, PCoA differentiated bacterial communities between partial and whole crop peas (Fig. 2). The relative abundances reported in the following were calculated on the basis of ASVs documented in Table S5 and Table S7. Native pea seeds were hardly colonized by bacterial or eukaryotic microorganisms.

In pea seed silages, *Lactobacillus* and *Pediococcus* spp. were most commonly identified (21 to 91% relative abundance) together with *Weissella*, *Bacillus*, and Staphylococcus spp. (maximal 54, 46, and 23% relative abundance, respectively).

Lactic acid bacteria (*Weissella*, *Pediococcus*, and *Lactobacillus* spp.) clearly dominated partial and whole crop pea silages throughout maturation (together 57 to 99% relative abundance). The remainder of epiphytic bacteria mainly comprised *Bacillus* spp. (maximal 33% relative abundance). *Lactobacillus* and *Pediococcus* spp. reached nearly 100% relative abundance after addition of LAB inoculant in most of the silages. On immature partial crop peas (BBCH 77), *Bacillus* and *Acetobacter* spp. were found with an abundance of 48 and 22%, respectively. Partial and whole crop peas at BBCH 79 harbored up to 44% *Weissella* spp. in addition to inoculated LAB.

After opening the silage, oxygen enters and aerobic microorganisms initialize deterioration (24, 25). Consumption of sugars and fermentation products raise silage temperature and the pH (24, 25). When the pH is increasing, *Bacillus* spp. as well as other aerobic bacteria and eukaryotes grow and further increase the temperature (25). Proliferation of molds completes silage deterioration, which results in dry matter (DM) loss, reduction of DM intake by animals when the silage is fed, and, in dairy applications, reduced milk yields (25). In the present study, aerobic storage did not clearly change the bacterial composition of pea silages (Fig. 2). *Lactobacillus* and *Weissella* spp. remained at up to 67 and 73% relative abundance on partial and whole crop peas, respectively. In seeds at BBCH 86, however, we found 81% to 98% relative abundance of Staphylococcus spp. after aerobic storage without artificial addition of LAB.

Application of silage additives, either chemicals or microorganisms, support fermentation before and, preferably, stability after opening of silages (25). Using the inoculant for ensiling led to bacterial communities dominated by *Lactobacillus* and *Weissella* spp. (up to 85 and 42% relative abundance, respectively, after silage opening). However, whole crop peas at BBCH 76 hosted 61% *Bacillus* and up to 39% *Solibacillus* spp., which matches previous observations (25). Dry matter concentrations of ensiled peas lower than 350 g/kg do not support rapid establishment of LAB in competition with other microorganisms and thus stable fermentation (26). In seeds harvested at BBCH 86 and ensiled with LAB inoculant, *Pediococcus* spp. remained at 38 to 51% after silage opening, while Staphylococcus spp. were still present with maximal 46% relative abundance.

Referring to eukaryotic communities, we predominantly found unclassified fungi (up to 99% relative abundance) and *Pichia* spp. (up to 86% relative abundance). *Pichia* spp. in silages are typically associated with high moisture contents and the presence of LAB as they ferment lactic acid (27). Next, the abundance of *Pichia* spp. seemed to decline throughout ongoing maturation of the peas. Aerobic storage clearly decreased the abundance of *Pichia* spp. in silages without inoculation with LAB strains to maximal 31%. Then, *Penicillium*, *Cladosporium*, and *Hyphopichia/Candida* clade spp. as well as unclassified representatives of the *Saccharomycetaceae*, *Sporidiobolaceae*, and Aspergillaceae families, were detected in individual samples. They primarily occurred in silages made from immature stages (i.e., BBCH 76 and 77) and after opening the silages. The presence of such eukaryotes is typical for spoiled silages (24, 27). The application of LAB inoculants can improve or impair aerobic stability of the silage, depending on the LAB strains used, the material, and the presence of yeasts that initiate aerobic deterioration (28). Reports on this are not consistent (28), but our results have shown that LAB inoculation widely inhibited the growth of eukaryotes under aerobic storage conditions.

### Changes in α-diversity.

**FIG 3:**
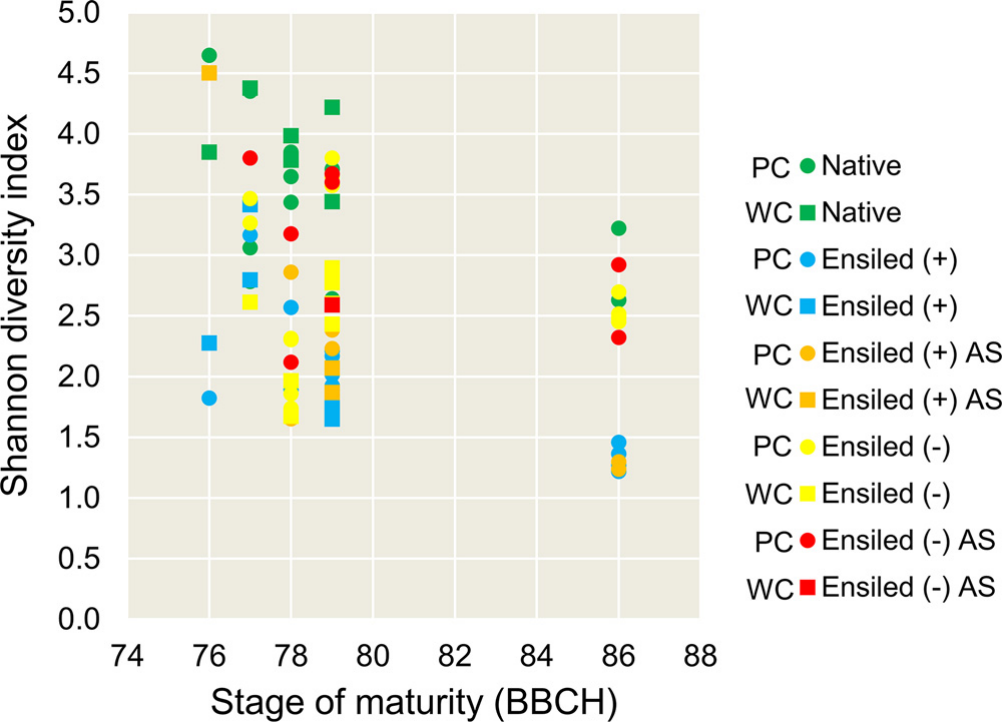
α-diversity of epiphytic bacterial communities on native and ensiled field peas based on the Shannon index discerned according to treatment and crop variant. BBCH maturity stages were assigned according to Meier (45) and are specified in Table 2. + denotes addition of microbial inoculant; – denotes ensiling without addition of inoculant; AS, aerobic storage (i.e., silages were stored 7 days under aerobic conditions); PC, partial crop peas; WC, whole crop peas.

Calculated Shannon diversity index values are shown in Fig. 3. The Kruskal-Wallis test revealed significant differences in α-diversity of bacteria among maturity stages (*P < *0.01) and treatments (*P < *0.001), whereas no differences were found between the crop variants (*P = *0.564). The Shannon index values generally decreased during ongoing maturation of the plants. Shannon diversity was highest in native peas and lowest in pea silages inoculated with LAB (Fig. 3). Aerobic storage of inoculated silages did not change Shannon diversity (Fig. 3). Pea silages made without LAB inoculant showed higher Shannon diversity, which further increased during aerobic storage (Fig. 3). Hence, application of inoculants supported stable bacterial communities dominated by LAB. This was most evident from the beginning of BBCH 79 when DM concentrations of the partial and whole crop peas were higher than 400 g/kg. In premature pea silages (BBCH 76 and 77), Shannon diversity was high even with LAB inoculation (Fig. 3). High water activity at these stages may support the growth of concomitant microorganisms in addition to natural and applied LAB (27, 29).

### Nutrient concentrations of pea maturity stages and ensiling quality traits.

The results of additional analyses of DM, crude nutrient, and detergent fiber concentrations of native and ensiled pea seeds, partial crop peas, and whole crop peas are summarized in Table S8.

Pea seeds ripened much faster than partial or whole crop peas (Table S8), which has to be considered during seed harvesting at premature stages with regard to harvesting technology. Harvest of seeds in which nutrient storage is complete but that are not fully ripened (from BBCH 79 at the latest to BBCH 86) may have phytosanitary benefits (30). Pea seeds had 457 g starch and 133 g sugar per kg DM at BBCH 76, and 519 g starch and 51 g sugar per kg DM at BBCH 86. Together with high protein concentrations in the seeds, which were more or less consistent within the tested maturity range (Table S8), this offers the opportunity to partly replace protein-rich feeds and cereals by peas in diets for ruminants and monogastric livestock (31–33).

Partial crop peas ranged in nutrient density and fiber concentration between pea seeds and whole crop peas (Table S8). They offer a balance in protein and fiber supply. In partial and whole crop peas, crude protein concentration was higher at late maturity (from BBCH 78 on; Table S8). This is usually similar to the concentration of starch (34, 35) and linked to pod development (34). Digestible nonstarch polysaccharides (mostly glucose and xylose residues) are a source of energy the plant provides in addition to energy provided by the seeds, but these components decrease during nutrient storage in the seeds and plant maturation (36).

On a DM basis, amino acid concentrations tended to be lower at later stages of maturity (Table S9). Ensiling modified amino acid composition, likely as a result of microbial degradation and synthesis (37).

Ensiling characteristics and the silages’ concentrations of ammonia, lactic acid, acetic acid, and alcohols are given in Table S10. Propionic acid, butyric acid, valeric acid, and caproic acid concentrations were mostly below detection limit.

The pea seed silages had an acidic, fresh scent and a moist doughy texture. Their pH values ranged from 4.2 to 5.9 and from 4.6 to 6.1 with and without LAB inoculant, respectively (Table S10). Silages from premature seeds (BBCH 76) were spoiled. After aerobic storage, the pH was higher than it was at the time of opening. Pea seeds ensiled at a late maturity stage (BBCH 86) had the highest pH values and lowest aerobic stability (Table S10). After aerobic storage, they were widely molded. Produced ammonia, acetic acid, and butanol levels were lower at late maturity (Table S10). In the pea seed silages, lactic acid was formed at BBCH 76 only after LAB inoculation. Also, at BBCH 78 and 79 more lactic acid was formed after using the additive. At BBCH 86, there was no lactic acid formation, either with or without LAB additive (Table S10). The concentrations of ammonia, acetic acid, and butanol were higher in seed silages without inoculant than with the addition of LAB until BBCH 79 (Table S10).

As with pea seeds, most of partial crop and whole crop pea silages were of good quality, with pH distinctly lower than 5.0. They had an acidic, nutty scent and a moist texture until BBCH 78. At BBCH 79 and 86, the silages were dry and strawlike. The concentration of ammonia was maximal 4.1 g/kg DM (i.e., 14% NH_3_-N of total nitrogen) and lowest at late maturity (Table S10). Ammonia is in silages mainly associated with reduction of palatability and intake depression (29, 38). In silages from partial and whole crop peas, the concentrations of lactic acid showed a decreasing tendency with increasing maturity (Table S10). In most cases, the lactic acid concentration differed only slightly between silages with and without LAB additive. In some cases, even more lactic acid was formed without LAB additive, which shows the significant presence of natural LAB. Significant quantities of butanol were detected in partial and whole crop pea silages (Table S10). Butanol concentration was low when DM concentration was high. Partial and whole crop pea silages at BBCH 77 and higher were stable for 104 to 168 h under aerobic storage conditions (Table S10). Despite LAB inoculation, silages made from premature materials (BBCH 76) were often mildewed after aerobic storage and had a perceptible scent of ammonia. Growth rates of spoiling bacteria increase along with availability of free water (39), and thus pea plants having less than 350 g DM per kg should be wilted before ensiling to maintain stable lactic acid fermentation (26).

Apart from DM concentration, sugar concentration and the sugar-to-buffering capacity ratio influence pH during ensiling and the appearance of LAB (40). In the native pea crop variants, total sugar concentration ranged from 24 to 76 g/kg DM. It was higher in the premature stages (BBCH 76 to 78) than in ripened materials. Ensiling generally reduced sugar concentration (7 to 37 g/kg DM), because oligomeric sugars are a substrate for lactic acid fermentation (10). In mature seed silages (BBCH 86), no sugar fermentation occurred (36 g/kg DM in native seeds, 36 g/kg DM in control silages, and 21 g/kg DM in silages with added inoculant, respectively), which corresponded to the stable pH (Table S10).

The current study did not consider year, site, or cultivar effects, which undoubtedly contribute to variation in canopy structure and development. However, we summarized results of the study relevant for practice to give an idea how harvesting and preserving of field peas can be controlled and improved (Table 1). The German Agricultural Society (DLG) key for evaluating roughages (41) was used to classify the ensiling success. This considers the measured proportion of acetic acid, butyric acid, and the pH value depending on the DM concentration. As a result, all silages achieved at least 85 out of 100 points and are rated as “good” to “very good.” This excludes silages that were obviously spoiled (e.g., seed silages at BBCH 76). However, the critical NH_3_-N content, which, according to the DLG key (42), should be a maximum of 10% of the total nitrogen, was often exceeded until BBCH 77, especially in silages that were produced without silage additives. With regard to the preservation of true protein and the protein solubility in the rumen, deamination and the associated formation of biogenic amines must be limited (43). Therefore, dry silage with a DM concentration of more than 500 g/kg is a sensible option for conservation, at least in case of partial or whole crop peas. In such a silage, the fermentation activity of the epiphytic LAB is alone not sufficient to achieve a preservative effect, due to the osmotic conditions. It is rather the combination of dryness and the anaerobic environment that is conserving the material, supported by lactic acid production.

**TABLE 1:** Prospectively relevant information for practice on harvesting and preserving field peas based on the results of this study

Stage of maturity at harvest (BBCH)^*a*^	76	77	78	79	86
Dry matter concn at harvest (g/kg)					
Seeds	310	390	430	550	740
Partial crops	250	300	340	420	630
Whole crops	250	300	360	450	590
Nutrient storage in seeds	incomplete	incomplete	almost complete	complete	complete
Preservation by ensiling reasonable					
Seeds	limited	limited	yes	yes	yes
Partial and whole crops	limited	limited	yes	yes	yes
Use of silage additives required					
Seeds	yes	yes	yes	yes	yes
Partial and whole crops	yes	yes	not mandatory	not mandatory	not mandatory
Silage quality to expect^*b*^					
Seeds	low		high	high	limited^*c*^
Partial and whole crops	low	limited^*c*^	high	high	high
Aerobic stress tolerance^*d*^					
Seeds			high	high	low^*c*^
Partial and whole crops	low^*c*^	high	high	high	high

a Maturity stages are encoded using the BBCH code for phenological maturity of plants according to Meier (45).

b Classified according to DLG keys (55, 56) on the basis of acetic acid, butyric acid, and NH_3_-N concentration; pH value; and aerobic stability.

c Depends on use of silage additive.

d Assuming a minimum of 72 h of aerobic stability is sufficient for practical application. This, however, depends on farm size and management.

### Conclusions.

The present study has shown that pea seeds were generally little colonized by epiphytic microorganisms. To produce stable silages from pea seeds and to protect them from spoilage after opening, the use of silage additives is required. As such, LAB inoculants can replace natural LAB. In the case of partial and whole crop peas, a significant natural stock of LAB was established during plant maturity. Our results suggest that partial and whole crop peas can be successfully ensiled beginning at BBCH 78 even without the use of inoculants. This could contribute to a reduction in farm costs and increased cultivation of field peas as feed plants. At BBCH 78, nutrient storage in the seeds is almost complete. Significant loss of nutrients is therefore not expected with harvest of pea plants from this stage on. Then, partial crop peas are of particular interest for ruminant nutrition, as they add easily soluble nutrients (protein and starch) as well as fiber to the ration.

## MATERIALS AND METHODS

### Harvesting and ensiling of peas.

**FIG 4:**
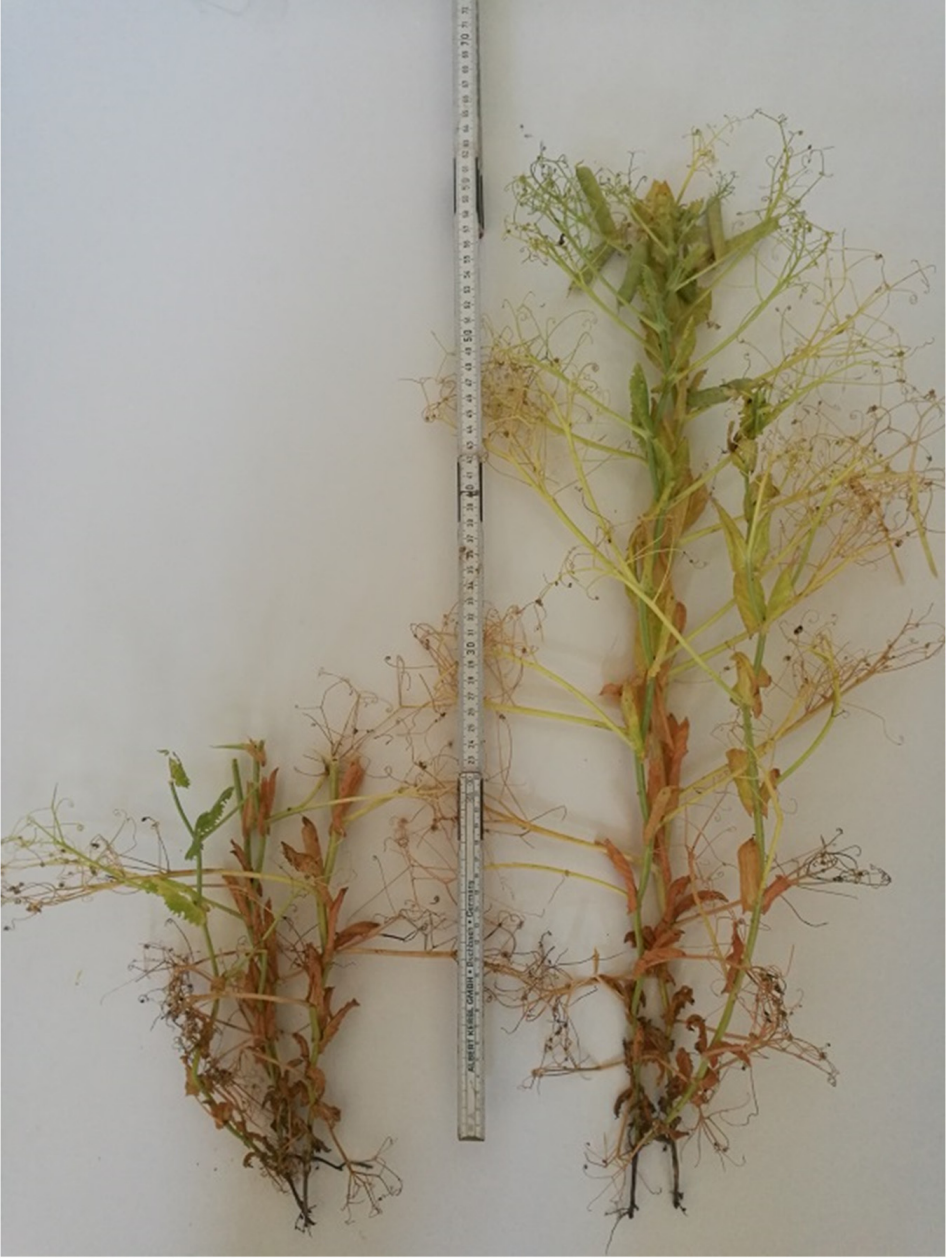
Partial crop of the pea cultivar “Astronaute” was harvested at approximately 25 cm height (beneath the lowest pods; on this photograph with a maturity referring to BBCH 79); specification of the maturity stage is given in Table 2; photo by C. Kuhnitzsch.

The field pea cultivar “Astronaute” (Fig. 4) was grown and harvested in Köllitsch (Saxony, Germany) in 2018. The cultivar “Astronaute” was chosen because it is one of the most widely used cultivars in Saxony and nationwide in both conventional and organic farming. Specific information on phenological, morphological, and yield characteristics can be obtained online from the Federal Plant Variety Office (44). Representative spots were sampled by hand in June and July at five maturity stages on the basis of the seeds’ DM concentration. At each of these stages, material was collected representing the seeds, partial crops, and whole crops. The investigator additionally determined phenological characteristics of the plants at each stage of maturity and classified them in the BBCH scaling (45). The DM concentrations at individual stages and corresponding BBCH codes are given in Table 2. Whole crops were cut 10 cm above the ground, whereas partial crops were cut at 25 cm height (Fig. 4), which means that approximately 58% (based on plant height) of the upper part of the plant was harvested. Strict use of gloves, cleaning, and disinfection of the equipment avoided contamination of the samples. From the native seeds and crops, Rostock Model Silages were prepared according to Hoedtke and Zeyner (46). Initial DM concentrations were those given in Table 2. The materials were chopped, vacuum-sealed in polyethylene bags (three bags per maturity stage for seeds, partial crops, and whole crops, respectively, with 2 kg material each), and stored at approximately 25°C for a minimum of 59 and maximum of 62 days. Two silage variants were prepared: (i) a control without any inoculant, and (ii) with addition of homofermentative LAB (Lactobacillus plantarum LSI NCIMB 30083 1k20736 and L256 NCIMB 30084 1k20737, and Pediococcus acidilactici P11 DSM 23689 1k1011 and P6 DSM 23688 1k1010 strains; together 1.0 × 10^11^ CFU per gram fresh matter) and carbohydrate degrading enzymes using a commercial preparation (Josilac classic; Josera GmbH & Co. KG, Kleinheubach, Germany). This LAB preparation was chosen because it is widely used in Germany, has a high practical relevance, is also approved for organic farms, and covers a wide DM range (25 to 40%). The type of the enzymes included in this preparation is not known and is confidential to the manufacturer.

**TABLE 2:** Dry matter concentration of pea seeds, partial crop peas, and whole crop peas at harvest at five maturity stages

Maturity (BBCH)^*a*^	Crop variant	Dry matter (g/kg)
76	Seeds	313
	Partial crops	251
	Whole crops	249
77	Seeds	389
	Partial crops	305
	Whole crops	301
78	Seeds	427
	Partial crops	341
	Whole crops	363
79	Seeds	549
	Partial crops	421
	Whole crops	447
86	Seeds	737
	Partial crops	632
	Whole crops	591

a Maturity stages are encoded using the BBCH code for phenological maturity of plants according to Meier (45).

### DNA extraction.

A quantity of 5 g sample material previously chopped and mixed was incubated for 15 min in 100 mL suspension consisting of 0.58 g NaH_2_PO_4_ × 2 H_2_O, 2.5 g Na_2_HPO_4_ × 2 H_2_O, 4 g NaCl, 1 g tryptic peptone, and 0.3 mL Tween 80 in 1 L water, with a pH of 7.0 as proposed by the Association of German Agricultural Analytic and Research Institutes (VDLUFA) (47). The sample material and the suspension were homogenized for 5 min at 230 rpm using a Seward Stomacher 400 Circulator paddle blender (Seward Ltd., Worthing, United Kingdom). The resulting liquid phase was centrifuged at 18,000 × *g* for 5 min, transferred to 2 mL tubes, and centrifuged at 10,000 × *g* for 1 min. Genomic DNA was extracted and purified using the *Quick*-DNA Fungal/Bacterial Miniprep Kit (Zymo Research Corp., Irvine, CA, USA) following the manufacturer’s instructions. The DNA concentration was determined using the Invitrogen Qubit 3.0 fluorometer and the Qubit dsDNA BR assay kit (Thermo Fisher Scientific Inc., Waltham, MA, USA). Extracted DNA was stored at −20°C.

### PCR amplification and amplicon sequencing.

The PCR and purification of the PCR products were carried out in accordance with the instructions provided for the Illumina MiSeq System (48). The amplification of the V3–V4 region of 16S rRNA genes was performed on a PCR SensoQuest Labcycler (SensoQuest GmbH, Göttingen, Germany) using the forward primer V3f (5′-CCTACGGGNGGCWGCAG-3′) and the reverse primer V4r (5′-GGACTACHVGGGTATCTAATCC-3′) (49). For amplification of the V8–V9 region of 18S rRNA genes, the forward primer 1422f (5′-ATAACAGGTCTGTGATGCCCT-3′´) and the reverse primer 1797r (5′-GCCTCCYGCAGGTTCACCTAC-3′) (50) were used. Each sample of 2.5 μL microbial DNA (5 ng/μL in 10 mM Tris-HCl, pH 8.5) was mixed with PCR master mix, consisting of 1 μL each of the barcoded forward and reverse primer (10 pmol/μL; Eurofins Genomics Germany GmbH, Ebersberg, Germany), 12.5 μL of 2 × KAPA HiFi DNA polymerase (Hot Start Ready Mix; KAPA Biosystems Inc., F. Hoffmann-La Roche AG, Basel, Switzerland), and 8 μL of PCR grade water. The PCR was performed under the following conditions: hot start at 95°C for 3 min, followed by 25 cycles of 95, 55, and 72°C, each for 30 s, and a final extension at 72°C for 5 min, then hold at 4°C. The PCR products were analyzed by gel electrophoresis in 1.5% agarose. PCR cleanup was carried out using AMPure XP beads (Beckman Coulter Genomics Inc., Chaska, MN, USA; 20 μL per sample) and a 96-well 0.2 mL PCR plate (Bio-Rad Laboratories Inc., Hercules, CA, USA). Index PCR was performed using index primers of the Nextera XT Index kit (Illumina Inc., San Diego, CA, USA). The library was arranged on a TruSeq Index Plate Fixture (Illumina Inc., San Diego, CA, USA) as given in the manual (48). Sequencing of the amplicons was performed on the Illumina MiSeq system (Illumina Inc., San Diego, CA, USA) using the MiSeq reagent kit v3 (600-cycle), followed by demultiplexing and removal of barcode sequences by the Illumina software.

### Chemical analyses.

Dry matter (after freeze-drying), crude ash, crude protein, acid ether extract, starch (determination by polarimetry), and sugars (determination by Luff-Schoorl method), crude fiber, and detergent fibers were analyzed according to VDLUFA (47) using the methods 3.1, 4.1.1, 5.1.1 B, 6.1.1, 6.5.1, 6.5.2, 6.5.3, 7.1.1, 7.2.1, and 8.1, respectively. The neutral detergent fiber was determined after pretreatment with heat stable amylase. Neutral detergent fiber and acid detergent fiber were expressed exclusive of residual ash. The proteins were hydrolyzed with hydrochloric acid, and individual amino acids were analyzed according to VDLUFA (47) method no. 4.11.1 using a Biochrom 30 Amino Acid Analyser with PEEK-Sodium Prewash Column (100 mm × 4.6 mm) and PEEK-Oxidized Feedstuff Column (200 mm × 4.6 mm) (Biochrom Ltd., Cambridge, United Kingdom). For detection of tryptophan, the proteins were hydrolyzed with phosphoric acid and hydrochloric acid. Tryptophan was analyzed according to Fontaine et al. (51) by liquid chromatography (Agilent 1100 Series fitted with 150 mm × 4.6 mm × 5 μm ZORBAX Eclipse XDB-C8 column; Agilent Technologies Inc., Santa Clara, CA, USA). Lactic acid concentrations were determined using liquid chromatography (internal method LKS FMUAA 166; the laboratory was accredited according to DIN EN ISO/IEC 17025:2018). Short chain fatty acids (SCFA) and alcohols produced during the fermentation of pea silages were determined after aqueous extraction by gas chromatography using a Shimadzu GC2010 (Shimadzu Corp., Kyoto, Japan) with flame ionization detector. An SGE BP21 separation column (30 m × 0.53 mm × 0.5 μm) (Trajan Scientific and Medical, Ringwood, AU-VIC, Australia) was used. The extracts were centrifuged at 2,000 × *g* before injection. The following settings were used for detection of SCFA: on-column injection, 0.5 μL injection volume, 180°C injection temperature, constant pressure of 22.7 kPa (i.e., 29.7 cm/s linear velocity and 3.64 mL/min column flow), 85°C initial oven temperature, raised up by 8°C/min to 200°C and held for 6 min, and 200°C detection temperature; and for detection of alcohols: on-column injection, 0.5 μL injection volume, 180°C injection temperature, constant column flow of 7.7 mL/min, 35°C initial oven temperature held for 2.5 min, raised up by 8°C/min to 50°C, then by 100°C/min to 200°C and held for 2 min, and 200°C detection temperature. Helium was the carrier and makeup gas. The concentration of target analytes was determined on the basis of an external standard calibration. The aerobic stability of the silages was tested following the procedure of Honig (52) and was expressed as time until the temperature difference between material (silage) and environment exceeds 3 K. The ammonia concentrations were determined according to the method of Conway and Byrne (53).

### Bioinformatic and statistical analysis.

Bioinformatic analysis of MiSeq amplicon sequences was performed with QIIME 2 version 2019.1 (54) including removal of primers by Cutadapt, quality and length filtering, chimera removal, DADA2 clustering, and taxonomic assignment using the SILVA 132 rRNA database (55, 56). The available number of sequences was normalized among samples by rarefaction, where sampling depth was restricted to 20,000 reads in the 16S rRNA data set and to 12,000 reads in the 18S rRNA data set. Amplicon sequence variants (ASVs) were used for all further calculations and statistical tests. The 16S rRNA ASVs that were assigned to archaea, chloroplasts, or mitochondria were removed from the 16S rRNA data set. The archaea were removed because the used primers do not have reliable coverage (49). The 18S rRNA ASVs assigned to the pea plant itself (i.e., to the phylum *Charophyta*) were removed from the 18S rRNA data set. Two-way PERMANOVA and a principal coordinate analysis (PCoA) were performed in PAST version 4.01 (57) based on Bray-Curtis similarity considering crop variant and stage of maturity (for native peas) or crop variant and treatment (for pea silages). The Shannon diversity index was calculated using QIIME 2. The Kruskal-Wallis test was used in SAS version 9.4 NPAR1WAY (SAS Institute Inc., Cary, NC, USA) to identify differences in α-diversity (Shannon index) among maturity stages, crop variants, or treatments. The significance level for all statistical tests was set to *P < *0.05. Statistical tests were not performed with data referring to pea seeds (16S rRNA and 18S rRNA) and partial and whole crop peas (18S rRNA), because the number of reads available after filtering (see above) did not reveal notable microbial colonization of native materials.

### Data availability.

Raw sequence data were deposited at the European Nucleotide Archive (ENA) under the study accession number PRJEB45910.
