# Supplementary material for: Changes in Composition and Diversity of Epiphytic Microorganisms on Field Pea Seeds, Partial Crop Peas, and Whole Crop Peas during Maturation and Ensiling with or without Lactic Acid Bacteria Inoculant
Source: Microbiol Spectr. 2022 Aug 10;10(4):e00953-22. doi: 10.1128/spectrum.00953-22 (PMC9431205; doi:10.1128/spectrum.00953-22)
Supplement: Supplemental file 1 — Fig. S1 and Tables S1 to S4, S6, and S8 to S10. Download spectrum.00953-22-s0001.pdf, PDF file, 0.9 MB [file spectrum.00953-22-s0001.pdf]

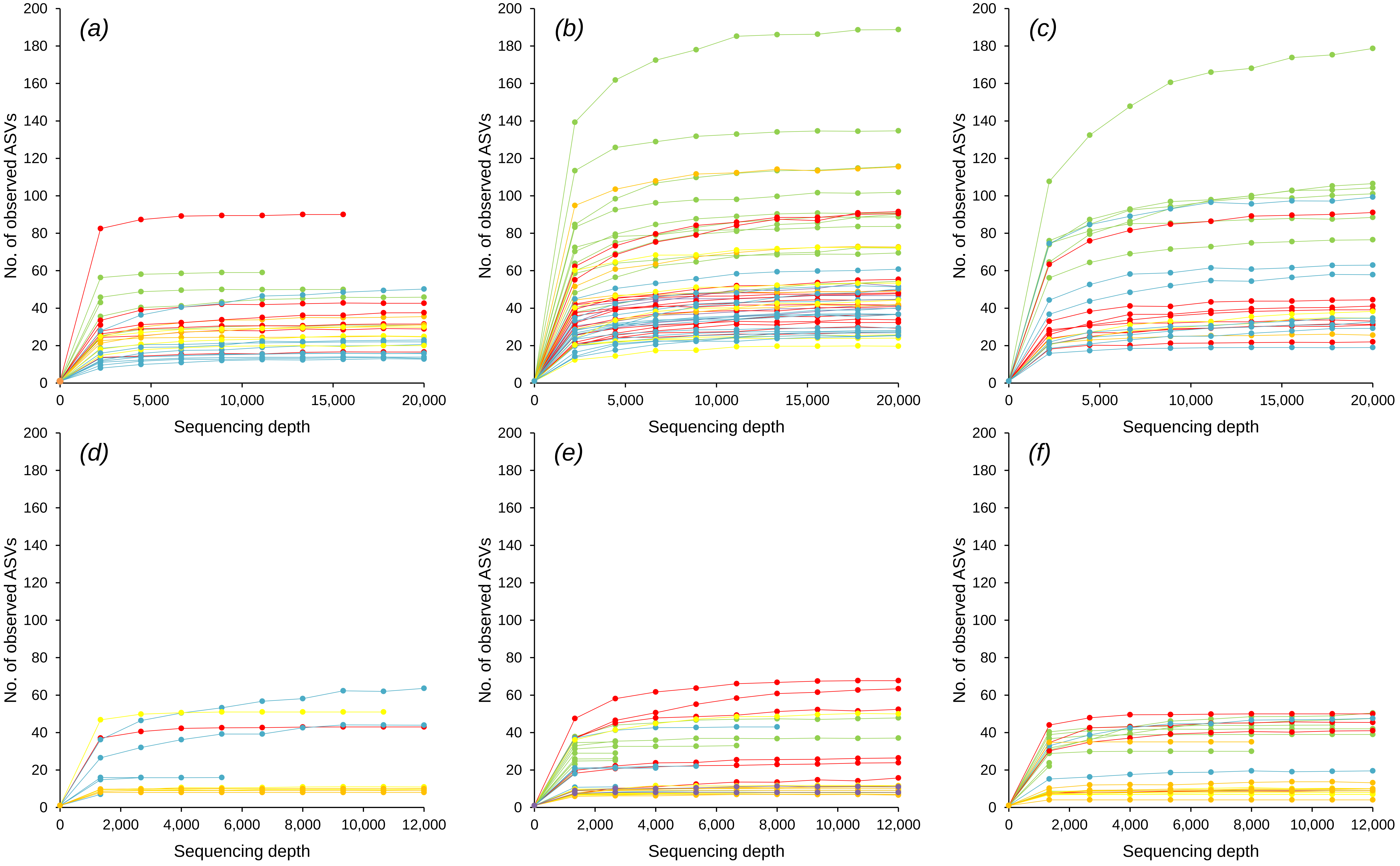

Figure S1. Rarefaction curves of 16S rRNA reads in (a) pea seeds, (b) partial crop peas, (c) whole crop peas, and 18S rRNA reads in (d) pea seeds, (e) partial crop peas, and (f) whole crop peas, discerned according to treatment (native peas: green; ensiled (+) peas: blue; ensiled (+) peas after 7 d aerobic storage: yellow; ensiled (–) peas: red; ensiled (–) peas after 7 d aerobic storage: orange). (+) denotes addition of microbial inoculant, (–) denotes ensiling without addition of inoculant. The rarefaction curves represent the number of amplicon sequence variants (ASVs) as a function of sequencing depth (number of reads). Each point represents a mean of 10 iterations. The standard deviation ranged from 0 to 5.08 (16S rRNA) and from 0 to 3.89 ASVs (18S rRNA).

Table S1. Number of observed amplicon sequence variants (ASVs) from 16S rRNA analysis.

ID	Maturity stage (BBCH)	Plant part	Treatment	Observed ASVs
SC1	76	Seeds	Native	23
SC2	76	Seeds	Native	59
SA67	76	Partial crop	Native	104
SA68	76	Partial crop	(+)	54
SC3	76	Whole crop	Native	78
SC4	76	Whole crop	(+)	64
SC5	76	Whole crop	(+) AS	118
SC6	77	Seeds	Native	20
SC7	77	Seeds	Native	24
SC10	77	Partial crop	Native	93
SC11	77	Partial crop	Native	84
SC12	77	Partial crop	Native	70
SC14	77	Partial crop	(+)	38
SA21	77	Partial crop	(-)	106
SC13	77	Partial crop	(-)	95
SA22	77	Partial crop	(-) AS	75
SC8	77	Whole crop	Native	89
SA70	77	Whole crop	(+)	65
SC9	77	Whole crop	(+)	103
SA69	77	Whole crop	(-)	96
SA71	78	Seeds	Native	15
SC15	78	Seeds	Native	11
SC17	78	Seeds	(+)	17
SC18	78	Seeds	(+)	16
SC16	78	Seeds	(-)	34
SA75	78	Partial crop	Native	117
SA76	78	Partial crop	Native	135
SA77	78	Partial crop	Native	193
SA32	78	Partial crop	(+)	65
SA33	78	Partial crop	(+)	39
SA34	78	Partial crop	(+)	47
SA36	78	Partial crop	(+)	38
SA35	78	Partial crop	(+) AS	54
SA37	78	Partial crop	(+) AS	75
SA17	78	Partial crop	(-)	35
SA30	78	Partial crop	(-)	45
SA31	78	Partial crop	(-)	33
SA78	78	Partial crop	(-)	28
SA18	78	Partial crop	(-) AS	59
SA29	78	Partial crop	(-) AS	46
SA72	78	Whole crop	Native	112
SA73	78	Whole crop	Native	103
SC19	78	Whole crop	(-)	22
SC20	78	Whole crop	(-)	22
SA79	79	Seeds	Native	31
SA80	79	Seeds	Native	46
SA43	79	Seeds	(+)	14
SA44	79	Seeds	(+)	16

SA45	79	Seeds	(+)	14
SA38	79	Seeds	(-)	32
SA41	79	Seeds	(-)	31
SA42	79	Seeds	(-)	43
SA15	79	Partial crop	Native	80
SA16	79	Partial crop	Native	98
SA27	79	Partial crop	(+)	46
SA51	79	Partial crop	(+)	28
SA52	79	Partial crop	(+)	30
SA54	79	Partial crop	(+)	29
SA53	79	Partial crop	(+) AS	26
SA55	79	Partial crop	(+) AS	24
SA50	79	Partial crop	(-)	50
SA57	79	Partial crop	(-)	49
SA59	79	Partial crop	(-)	45
SA60	79	Partial crop	(-)	42
SA58	79	Partial crop	(-) AS	49
SA62	79	Partial crop	(-) AS	50
SA25	79	Whole crop	Native	193
SA26	79	Whole crop	Native	123
SA11	79	Whole crop	(+)	39
SA13	79	Whole crop	(+)	39
SA14	79	Whole crop	(+)	40
SA20	79	Whole crop	(+)	19
SA10	79	Whole crop	(+) AS	41
SA12	79	Whole crop	(+) AS	42
SA19	79	Whole crop	(-)	42
SA46	79	Whole crop	(-)	42
SA48	79	Whole crop	(-)	46
SA49	79	Whole crop	(-)	32
SA61	79	Whole crop	(-) AS	33
SA47	79	Whole crop	(-) AS	27
SA81	86	Seeds	Native	50
SA82	86	Seeds	Native	43
SA86	86	Seeds	(+)	15
SA88	86	Seeds	(+)	21
SA90	86	Seeds	(+)	55
SA92	86	Seeds	(+)	24
SA87	86	Seeds	(+) AS	30
SA89	86	Seeds	(+) AS	26
SA91	86	Seeds	(+) AS	20
SA40	86	Seeds	(-)	90
SA84	86	Seeds	(-)	39
SA85	86	Seeds	(-)	31
SA39	86	Seeds	(-) AS	36
SA63	86	Seeds	(-) AS	32
SA83	86	Seeds	(-) AS	25
SA93	86	Partial crop	Native	58
SA94	86	Partial crop	Native	92
SA4	86	Partial crop	(+)	29

SA5	86	Partial crop	(+)	30
SA6	86	Partial crop	(+)	67
SA8	86	Partial crop	(+)	31
SA28	86	Partial crop	(+) AS	49
SA7	86	Partial crop	(+) AS	22
SA2	86	Partial crop	(-)	44
SA3	86	Partial crop	(-)	52
SA9	86	Partial crop	(-)	37
SA95	86	Partial crop	(-)	61
SA1	86	Partial crop	(-) AS	47
SA64	86	Partial crop	(-) AS	45

(+) ensiling with use of bacterial inoculant, (-) ensiling without use of inoculant,
AS = aerobic storage (i.e., silages were stored 7 d under aerobic conditions).
Maturity stages are encoded according to Meier (45).

Table S2. Number of observed amplicon sequence variants (ASVs) from 18S rRNA analysis.

ID	Maturity stage (BBCH)	Plant part	Treatment	Observed ASVs
SD29	76	Seeds	Native	1
SD30	76	Seeds	Native	8
SD53	76	Seeds	(+)	8
SD6	76	Seeds	(+)	5
SD10	76	Partial crop	Native	25
SD11	76	Partial crop	Native	11
SD12	76	Partial crop	Native	10
SD1	76	Partial crop	(+) AS	12
SD38	76	Partial crop	(+) AS	8
SD13	76	Partial crop	(-) AS	9
SD37	76	Partial crop	(-) AS	10
SD33	76	Whole crop	Native	24
SD7	76	Whole crop	Native	30
SD8	76	Whole crop	Native	29
SD54	76	Whole crop	(+)	21
SD36	76	Whole crop	(+) AS	11
SD9	76	Whole crop	(+) AS	9
SD34	76	Whole crop	(-) AS	10
SD35	76	Whole crop	(-) AS	10
SD2	77	Seeds	Native	3
SD3	77	Seeds	Native	4
SD15	77	Partial crop	Native	23
SD16	77	Partial crop	Native	13
SD40	77	Partial crop	Native	25
SC66	77	Partial crop	(+)	11
SD26	77	Partial crop	(+)	9
SD25	77	Partial crop	(+) AS	7
SC56	77	Partial crop	(-)	24
SC65	77	Partial crop	(-)	55
SD41	77	Partial crop	(-) AS	7
SD42	77	Partial crop	(-) AS	11
SD4	77	Whole crop	Native	12
SD5	77	Whole crop	Native	22
SD81	77	Whole crop	Native	9
SC24	77	Whole crop	(+)	49
SD14	77	Whole crop	(+) AS	7
SD32	77	Whole crop	(+) AS	8
SB21	77	Whole crop	(-)	46
SC23	77	Whole crop	(-)	42
SD31	77	Whole crop	(-) AS	4
SD39	77	Whole crop	(-) AS	9
SD23	78	Seeds	Native	2
SD24	78	Seeds	Native	5
SD89	78	Seeds	Native	4

SA70	78	Seeds	(+)	16
SA72	78	Seeds	(+)	52
SA73	78	Seeds	(+)	75
SD28	78	Seeds	(+)	7
SA71	78	Seeds	(+) AS	51
SB22	78	Seeds	(-)	43
SD51	78	Seeds	(-)	7
SD56	78	Seeds	(-)	2
SD91	78	Seeds	(-)	5
SD17	78	Partial crop	Native	26
SD18	78	Partial crop	Native	29
SD19	78	Partial crop	Native	35
SA79	78	Partial crop	(+)	22
SA80	78	Partial crop	(+)	43
SD47	78	Partial crop	(+) AS	8
SD48	78	Partial crop	(+) AS	8
SB25	78	Partial crop	(-)	69
SD45	78	Partial crop	(-) AS	12
SD46	78	Partial crop	(-) AS	7
SA74	78	Whole crop	Native	50
SA75	78	Whole crop	Native	54
SB23	78	Whole crop	Native	44
SD44	78	Whole crop	(+) AS	7
SB24	78	Whole crop	(-)	50
SD82	78	Whole crop	(-) AS	35
SD88	78	Whole crop	(-) AS	14
SD49	79	Seeds	Native	5
SD50	79	Seeds	Native	9
SA81	79	Seeds	(+)	5
SD86	79	Seeds	(+)	16
SD87	79	Seeds	(+)	5
SD83	79	Seeds	(-)	11
SD84	79	Seeds	(-)	7
SD85	79	Seeds	(-)	10
SB28	79	Partial crop	Native	48
SD80	79	Partial crop	Native	37
SD92	79	Whole crop	Native	42
SD93	79	Whole crop	Native	39
SD61	86	Seeds	Native	7
SD62	86	Seeds	Native	6
SD65	86	Seeds	(+)	4
SD67	86	Seeds	(+)	9
SD69	86	Seeds	(+)	7
SD79	86	Seeds	(+)	3
SD66	86	Seeds	(+) AS	11
SD68	86	Seeds	(+) AS	10

SD90	86	Seeds	(+) AS	10
SD63	86	Seeds	(-)	9
SD74	86	Seeds	(-)	5
SD76	86	Seeds	(-)	2
SD78	86	Seeds	(-)	4
SD73	86	Seeds	(-) AS	8
SD75	86	Seeds	(-) AS	10
SD77	86	Seeds	(-) AS	9
SD70	86	Partial crop	Native	33
SD71	86	Partial crop	Native	19
SD94	86	Partial crop	(+)	18
SD96	86	Partial crop	(+)	21
SA82	86	Partial crop	(+) AS	51
SD95	86	Partial crop	(+) AS	14
SA83	86	Partial crop	(-)	69
SD58	86	Partial crop	(-)	8
SD64	86	Partial crop	(-)	17
SD72	86	Partial crop	(-)	27
SD57	86	Partial crop	(-) AS	8
SD59	86	Partial crop	(-) AS	12

(+) ensiling with use of bacterial inoculant, (-) ensiling without use of inoculant,
AS = aerobic storage (i.e., silages were stored 7 d under aerobic conditions).
Maturity stages are encoded according to Meier (45).

Table S3. Number of 16S rRNA reads per sample.

ID	Maturity stage (BBCH)	Plant part	Treatment	Reads per sample
SC1	76	Seeds	Native	783
SC2	76	Seeds	Native	12543
SA67	76	Partial crop	Native	52444
SA68	76	Partial crop	(+)	76170
SC3	76	Whole crop	Native	63343
SC4	76	Whole crop	(+)	68725
SC5	76	Whole crop	(+) AS	72056
SC6	77	Seeds	Native	982
SC7	77	Seeds	Native	2426
SC10	77	Partial crop	Native	46360
SC11	77	Partial crop	Native	22778
SC12	77	Partial crop	Native	46984
SC14	77	Partial crop	(+)	90167
SA21	77	Partial crop	(-)	309264
SC13	77	Partial crop	(-)	89765
SA22	77	Partial crop	(-) AS	124414
SC8	77	Whole crop	Native	42085
SA70	77	Whole crop	(+)	127435
SC9	77	Whole crop	(+)	87978
SA69	77	Whole crop	(-)	82166
SA71	78	Seeds	Native	859
SC15	78	Seeds	Native	355
SC17	78	Seeds	(+)	54368
SC18	78	Seeds	(+)	62429
SC16	78	Seeds	(-)	74208
SA75	78	Partial crop	Native	52144
SA76	78	Partial crop	Native	37015
SA77	78	Partial crop	Native	59392
SA32	78	Partial crop	(+)	169370
SA33	78	Partial crop	(+)	92173
SA34	78	Partial crop	(+)	93887
SA36	78	Partial crop	(+)	70879
SA35	78	Partial crop	(+) AS	95216
SA37	78	Partial crop	(+) AS	102042
SA17	78	Partial crop	(-)	128556
SA30	78	Partial crop	(-)	345646
SA31	78	Partial crop	(-)	181764
SA78	78	Partial crop	(-)	100349
SA18	78	Partial crop	(-) AS	171625
SA29	78	Partial crop	(-) AS	198022
SA72	78	Whole crop	Native	84360
SA73	78	Whole crop	Native	50821
SC19	78	Whole crop	(-)	38060
SC20	78	Whole crop	(-)	44165
SA79	79	Seeds	Native	59470
SA80	79	Seeds	Native	26371
SA43	79	Seeds	(+)	110425
SA44	79	Seeds	(+)	73348

SA45	79	Seeds	(+)	91215
SA38	79	Seeds	(-)	107227
SA41	79	Seeds	(-)	98725
SA42	79	Seeds	(-)	63482
SA15	79	Partial crop	Native	173538
SA16	79	Partial crop	Native	155927
SA27	79	Partial crop	(+)	237667
SA51	79	Partial crop	(+)	111937
SA52	79	Partial crop	(+)	72000
SA54	79	Partial crop	(+)	118817
SA53	79	Partial crop	(+) AS	103359
SA55	79	Partial crop	(+) AS	99545
SA50	79	Partial crop	(-)	96517
SA57	79	Partial crop	(-)	101054
SA59	79	Partial crop	(-)	101025
SA60	79	Partial crop	(-)	76781
SA58	79	Partial crop	(-) AS	94763
SA62	79	Partial crop	(-) AS	111384
SA25	79	Whole crop	Native	155380
SA26	79	Whole crop	Native	206072
SA11	79	Whole crop	(+)	313164
SA13	79	Whole crop	(+)	162496
SA14	79	Whole crop	(+)	437064
SA20	79	Whole crop	(+)	66050
SA10	79	Whole crop	(+) AS	223750
SA12	79	Whole crop	(+) AS	190599
SA19	79	Whole crop	(-)	120447
SA46	79	Whole crop	(-)	104663
SA48	79	Whole crop	(-)	67143
SA49	79	Whole crop	(-)	91394
SA61	79	Whole crop	(-) AS	117410
SA47	79	Whole crop	(-) AS	78479
SA81	86	Seeds	Native	16367
SA82	86	Seeds	Native	4279
SA86	86	Seeds	(+)	76848
SA88	86	Seeds	(+)	86302
SA90	86	Seeds	(+)	122655
SA92	86	Seeds	(+)	78477
SA87	86	Seeds	(+) AS	77062
SA89	86	Seeds	(+) AS	98183
SA91	86	Seeds	(+) AS	65508
SA40	86	Seeds	(-)	17125
SA84	86	Seeds	(-)	86993
SA85	86	Seeds	(-)	2422
SA39	86	Seeds	(-) AS	81517
SA63	86	Seeds	(-) AS	67566
SA83	86	Seeds	(-) AS	32070
SA93	86	Partial crop	Native	84521
SA94	86	Partial crop	Native	75260
SA4	86	Partial crop	(+)	168672

SA5	86	Partial crop	(+)	236970
SA6	86	Partial crop	(+)	338286
SA8	86	Partial crop	(+)	179699
SA28	86	Partial crop	(+) AS	268507
SA7	86	Partial crop	(+) AS	275263
SA2	86	Partial crop	(-)	113187
SA3	86	Partial crop	(-)	132227
SA9	86	Partial crop	(-)	132243
SA95	86	Partial crop	(-)	86941
SA1	86	Partial crop	(-) AS	206299
SA64	86	Partial crop	(-) AS	101577

(+) ensiling with use of bacterial inoculant, (-) ensiling without use of inoculant,
AS = aerobic storage (i.e., silages were stored 7 d under aerobic conditions).
Maturity stages are encoded according to Meier (45).

Table S4. Number of 18S rRNA reads per sample.

ID	Maturity stage (BBCH)	Plant part	Treatment	Reads per sample
SD29	76	Seeds	Native	154
SD30	76	Seeds	Native	148
SD53	76	Seeds	(+)	842
SD6	76	Seeds	(+)	196
SD10	76	Partial crop	Native	3604
SD11	76	Partial crop	Native	604
SD12	76	Partial crop	Native	1052
SD1	76	Partial crop	(+) AS	61794
SD38	76	Partial crop	(+) AS	120084
SD13	76	Partial crop	(-) AS	79817
SD37	76	Partial crop	(-) AS	128350
SD33	76	Whole crop	Native	2354
SD7	76	Whole crop	Native	8352
SD8	76	Whole crop	Native	2493
SD54	76	Whole crop	(+)	101407
SD36	76	Whole crop	(+) AS	78590
SD9	76	Whole crop	(+) AS	44485
SD34	76	Whole crop	(-) AS	110600
SD35	76	Whole crop	(-) AS	90603
SD2	77	Seeds	Native	71
SD3	77	Seeds	Native	110
SD15	77	Partial crop	Native	1521
SD16	77	Partial crop	Native	614
SD40	77	Partial crop	Native	2401
SC66	77	Partial crop	(+)	3687
SD26	77	Partial crop	(+)	67273
SD25	77	Partial crop	(+) AS	105220
SC56	77	Partial crop	(-)	37624
SC65	77	Partial crop	(-)	78009
SD41	77	Partial crop	(-) AS	83080
SD42	77	Partial crop	(-) AS	102498
SD4	77	Whole crop	Native	758
SD5	77	Whole crop	Native	1611
SD81	77	Whole crop	Native	63817
SC24	77	Whole crop	(+)	61806
SD14	77	Whole crop	(+) AS	48760
SD32	77	Whole crop	(+) AS	35938
SB21	77	Whole crop	(-)	22116
SC23	77	Whole crop	(-)	48713
SD31	77	Whole crop	(-) AS	13927
SD39	77	Whole crop	(-) AS	75244
SD23	78	Seeds	Native	38
SD24	78	Seeds	Native	60
SD89	78	Seeds	Native	66

SA70	78	Seeds	(+)	5733
SA72	78	Seeds	(+)	152421
SA73	78	Seeds	(+)	219075
SD28	78	Seeds	(+)	1647
SA71	78	Seeds	(+) AS	11997
SB22	78	Seeds	(-)	17918
SD51	78	Seeds	(-)	941
SD56	78	Seeds	(-)	56
SD91	78	Seeds	(-)	119
SD17	78	Partial crop	Native	3059
SD18	78	Partial crop	Native	3658
SD19	78	Partial crop	Native	3813
SA79	78	Partial crop	(+)	5378
SA80	78	Partial crop	(+)	9240
SD47	78	Partial crop	(+) AS	66469
SD48	78	Partial crop	(+) AS	77127
SB25	78	Partial crop	(-)	38710
SD45	78	Partial crop	(-) AS	101710
SD46	78	Partial crop	(-) AS	81470
SA74	78	Whole crop	Native	83798
SA75	78	Whole crop	Native	98331
SB23	78	Whole crop	Native	11200
SD44	78	Whole crop	(+) AS	67146
SB24	78	Whole crop	(-)	14493
SD82	78	Whole crop	(-) AS	8097
SD88	78	Whole crop	(-) AS	59816
SD49	79	Seeds	Native	321
SD50	79	Seeds	Native	447
SA81	79	Seeds	(+)	110
SD86	79	Seeds	(+)	2801
SD87	79	Seeds	(+)	96
SD83	79	Seeds	(-)	427
SD84	79	Seeds	(-)	168
SD85	79	Seeds	(-)	6493
SB28	79	Partial crop	Native	33966
SD80	79	Partial crop	Native	14800
SD92	79	Whole crop	Native	16638
SD93	79	Whole crop	Native	13647
SD61	86	Seeds	Native	162
SD62	86	Seeds	Native	337
SD65	86	Seeds	(+)	106
SD67	86	Seeds	(+)	1031
SD69	86	Seeds	(+)	186
SD79	86	Seeds	(+)	75
SD66	86	Seeds	(+) AS	74578
SD68	86	Seeds	(+) AS	25555

SD90	86	Seeds	(+) AS	51599
SD63	86	Seeds	(-)	215
SD74	86	Seeds	(-)	96
SD76	86	Seeds	(-)	83
SD78	86	Seeds	(-)	167
SD73	86	Seeds	(-) AS	26772
SD75	86	Seeds	(-) AS	24820
SD77	86	Seeds	(-) AS	47036
SD70	86	Partial crop	Native	7607
SD71	86	Partial crop	Native	1489
SD94	86	Partial crop	(+)	2176
SD96	86	Partial crop	(+)	5215
SA82	86	Partial crop	(+) AS	26125
SD95	86	Partial crop	(+) AS	829
SA83	86	Partial crop	(-)	69089
SD58	86	Partial crop	(-)	211
SD64	86	Partial crop	(-)	98958
SD72	86	Partial crop	(-)	70548
SD57	86	Partial crop	(-) AS	101120
SD59	86	Partial crop	(-) AS	257309

(+) ensiling with use of bacterial inoculant, (-) ensiling without use of inoculant,
AS = aerobic storage (i.e., silages were stored 7 d under aerobic conditions).
Maturity stages are encoded according to Meier (45).

Table S6. Relative abundance of epiphytic bacteria on pea seeds, partial crop peas, and whole crop peas after harvesting (native material) at five maturity stages.

Maturity stage (BBCH)	Plant part	*Bacillus*	*Oceano-bacillus*	*Exiguo-bacterium*	*Entero-coccus*	*Lacto-bacillus*	*Pedio-coccus*	*Weissella*	*Hafnia-Obesum-bacterium*	*Serratia*	Uncl. (*Entero-bacteriaceae*)	*Pseudo-monas*	Others
76	Seeds	7.0 (0.94)	0.7 (1)	0	0.9 (0.3)	20.7 (6.76)	11.1 (1.85)	5.2 (2.9)	12.2 (2.12)	0	17.1 (7.37)	7.9 (7.0)	17.1
	Partial crops	26.7	6.0	0.7	10.4	5.4	0.3	0.2	3.8	4.9	34.2	0.2	7.3
	Whole crops	3.4	0.6	9.4	8.4	0.6	0.1	< 0.1	6.3	23.1	44.7	0.4	3.0
77	Seeds	16.7 (6.51)	2.2 (2.1)	0	3.3 (0.086)	28.8 (31.0)	7.2 (5.6)	0.3 (0.4)	0	0.6 (0.8)	11.1 (0.152)	16.4 (16.5)	13.5
	Partial crops	11.2 (11.0)	2.0 (2.0)	0.2 (0.03)	7.0 (7.7)	3.1 (3.0)	4.5 (3.5)	20.7 (35.0)	4.0 (2.4)	3.4 (1.8)	38.3 (17.4)	0.6 (0.4)	5.2
	Whole crops	6.2	0.7	1.9	10.5	1.2	0.4	0.2	11.4	14.1	43.8	1.2	8.4
78	Seeds	4.7 (6.6)	0	0	0	24.4 (7.82)	6.6 (3.8)	9.6 (5.7)	0	0	43.2 (21.4)	2.4 (3.5)	9.0
	Partial crops	0.2 (0.2)	< 0.1 (0.01)	1.4 (0.79)	8.9 (1.7)	0.8 (0.04)	0.5 (0.3)	0.6 (0.3)	1.2 (0.37)	4.2 (1.6)	68.2 (1.33)	5.0 (5.5)	9.3
	Whole crops	0.2 (0.2)	< 0.1 (0.01)	2.5 (0.87)	5.6 (3.5)	0.6 (0.2)	0.9 (1.0)	8.2 (6.6)	2.1 (2.3)	1.8 (1.7)	71.4 (1.75)	2.4 (1.8)	4.2
79	Seeds	45.4 (64.1)	0	0.5 (0.6)	0.1 (0.04)	0.5 (0.3)	0.2 (0.08)	0.3 (0.2)	< 0.1 (0.05)	0.7 (0.7)	48.4 (60.5)	2.6 (2.2)	1.3
	Partial crops	18.9 (26.6)	< 0.1 (0.003)	0.6 (0.3)	2.5 (0.65)	0.6 (0.03)	0.2 (0.07)	3.0 (3.2)	0.4 (0.4)	1.4 (0.17)	61.5 (17.9)	10.3 (4.62)	0.9
	Whole crops	0.5 (0.6)	< 0.1 (0.01)	3.0 (1.3)	2.6 (1.4)	0.6 (0.3)	0.4 (0.3)	2.2 (1.7)	2.0 (2.8)	0.8 (1.0)	75.1 (9.71)	7.8 (2.9)	5.0
86	Seeds	34.3 (47.3)	0	0.8 (0.9)	10.6 (1.68)	4.2 (1.5)	1.8 (1.7)	2.6 (0.79)	0.6 (0.4)	13.3 (15.8)	19.6 (18.7)	1.7 (1.7)	10.5
	Partial crops	0.3 (0.2)	< 0.1 (0.03)	1.2 (1.2)	4.8 (3.5)	0.4 (0.2)	0.2 (0.07)	7.8 (5.2)	0.1 (0.08)	0.3 (0.03)	73.6 (4.05)	9.6 (5.1)	1.8

Relative abundances based on amplicon sequence variants (ASVs) and are given as percent of total bacteria. Maturity stages are encoded according to Meier (45). In one case among the shown, *Enterobacteriaceae* could not be classified beyond the rank of family. Bacteria with relative abundance lower than 5% are added up under the term “others”. Analyses were performed using one sample per variant, unless otherwise indicated. Means of two biological replicates per variant are highlighted grey; means of three replicates per variant are highlighted blue. The standard deviation of means is given in brackets.

Table S8. Crude nutrient and detergent fiber composition of pea seeds, partial crop peas, and whole crop peas at harvesting (native material), after ensiling with addition of bacterial inoculant (+), and after ensiling without addition of inoculant (–), at five maturity stages.

Maturity (BBCH)	Crop variant	Treatment	DM	CA	CP	AEE	CF	NDF	ADF	ADL
76	Seeds	Native	294	35	231	14	77	103	98	6
		+	319	32	244	12	82	97	99	2
		–	287	36	272	16	93	110	116	3
	Partial crops	Native	257 (6.22)	53 (2.1)	196 (9.89)	10 (2.5)	135 (5.90)	188 (3.81)	165 (9.66)	14 (2.3)
		+	247	58	177	12	191	238	234	27
		–	254	57	173	15	193	242	210	26
	Whole crops	Native	256 (15.7)	87 (0.27)	129 (1.13)	14 (1.1)	283 (7.28)	359 (9.52)	315 (6.11)	45 (1.1)
		+	245	75	148	13	249	321	280	39
		–	236	83	149	12	291	337	323	46
77	Seeds	Native	385 (1.65)	28 (0.45)	228 (16.1)	8 (0.7)	58 (6.7)	97 (4.3)	75 (3.8)	1 (0.03)
	Partial crops	Native	312	66	171	12	210	266	236	31
		+	294	61	197	14	165	237	220	26
		–	286	61	178	13	152	237	236	24
	Whole crops	Native	308 (0.548)	73 (1.4)	147 (6.55)	9 (2)	241 (3.20)	309 (16.8)	276 (24.6)	40 (5.3)
		+	274	76	150	13	277	332	305	40
		–	279	72	150	13	264	314	265	37
78	Seeds	Native	401 (23.4)	31 (0.62)	207 (5.25)	15 (0.72)	63 (1.5)	161 (81.6)	99 (1.9)	2 (0.8)
		+	415 (0.446)	30 (0.17)	220 (2.41)	13 (3.6)	67 (3.6)	93 (14)	84 (4.4)	2 (0.05)
		–	404	32	229	12	65	89	85	1
	Partial crops	Native	325 (11.6)	62 (0.47)	158 (8.74)	18 (0.057)	198 (0.140)	273 (7.98)	233 (9.39)	37 (1.7)
		+	333	61	157	18	198	279	258	43
		–	321	56	160	16	168	230	287	46
	Whole crops	Native	347 (6.19)	72 (2.9)	120 (3.20)	15 (1.3)	281 (9.73)	365 (4.10)	312 (5.02)	47 (0.77)
		+	371	55	146	19	150	295	268	39
		–	329	76	133	20	320	393	328	46
79	Seeds	Native	542	31	225	15	62	132	78	11
		+	532	32	233	10	61	94	92	7
		–	523	32	240	12	69	102	82	5
	Partial crops	Native	432	52	186	14	120	150	150	13
		+	467	49	187	16	98	151	129	9
		–	458	49	187	15	101	137	119	9
	Whole crops	Native	426	69	173	15	161	223	239	32
		+	453	58	167	17	142	211	187	20
		–	433	66	161	16	156	202	206	24
86	Seeds	Native	733 (4.76)	31 (0.18)	224 (2.86)	13 (0.050)	63 (1.3)	96 (2.36)	105 (0.925)	7 (0.8)
		+	733	31	219	13	62	97	71	4
		–	740	32	227	12	64	112	69	6
	Partial crops	+	613	58	193	14	137	186	165	21
		–	550	67	170	13	175	247	188	26

ADF = acid detergent fiber, ADL = acid detergent lignin, AEE = acid ether extract, CA = crude ash, CF = crude fiber, CP = crude protein, DM = dry matter, n.a. = not analyzed (sample

material was not available for these analyses), NDF = neutral detergent fiber; NDF was assayed with amylase, ADF and NDF are expressed exclusive of residual ash.

DM is given in g/kg, all other analytes are given in g/kg DM. Maturity stages are specified in Table 1 and are encoded according to Meier (45). Analyses were performed using one sample per variant, unless otherwise indicated. Means of two biological replicates per variant are highlighted grey; means of three replicates per variant are highlighted blue; the standard deviation of means is given in brackets.

Table S9. Amino acid composition of pea seeds, partial crop peas, and whole crop peas at harvesting (native material), after ensiling with addition of bacterial inoculant (+), and after ensiling without addition of inoculant (–), at five maturity stages.

Maturity stage (BBCH)	Plant part	Treatment	Arg	Ile	Leu	Lys	Met	Phe	Thr	Trp	Val	Ala	Asp	Cys	Glu	Gly	Pro	Ser
76	Seeds	Native	28.2	9.2	15.7	14.5	1.9	9.9	9.3	2.0	10.4	13.3	22.8	2.2	36.9	8.6	8.4	10.7
		+	11.4	9.2	15.2	13.3	1.7	9.4	8.2	0.9	10.1	12.8	22.4	1.9	31.5	8.2	7.7	7.6
		–	9.0	10.1	16.8	11.9	1.8	10.1	6.3	1.4	11.5	16.0	17.3	1.6	29.4	9.2	8.5	6.1
	Partial crops	Native	15.1 (1.1)	6.0 (0.66)	10.2 (1.01)	8.4 (0.83)	1.4 (0.13)	6.2 (0.66)	7.0 (0.48)	1.7 (0.11)	6.9 (0.67)	8.0 (0.55)	18.4 (1.23)	1.4 (0.093)	21.6 (2.00)	6.0 (0.51)	5.8 (0.65)	7.3 (0.56)
		+	5.0	7.0	11.1	8.1	1.5	6.3	7.5	1.0	8.3	10.3	19.4	1.2	12.2	7.0	6.5	6.1
		–	3.6	7.0	11.2	7.5	1.5	6.7	7.5	1.0	8.1	10.0	16.9	1.1	7.7	6.8	6.4	5.3
	Whole crops	Native	7.8 (1.7)	4.2 (0.84)	7.1 (1.6)	5.3 (1.3)	1.1 (0.18)	4.4 (0.92)	8.2 (4.5)	1.3 (0.25)	5.0 (1.0)	5.4 (0.86)	12.7 (2.01)	0.9 (0.2)	12.7 (3.02)	4.6 (0.75)	4.4 (0.87)	4.8 (0.86)
		+	3.6	5.5	8.8	6.4	1.3	5.3	6.0	1.3	6.8	8.0	16.1	0.9	11.8	5.8	5.5	5.1
		–	2.1	5.6	8.9	5.0	1.3	5.1	5.6	1.3	6.5	8.1	9.8	0.8	6.0	5.9	5.1	4.5
77	Seeds	Native	21.9 (0.0360)	8.8 (0.23)	14.9 (0.388)	14.2 (0.279)	1.8 (0.093)	9.6 (0.17)	7.9 (0.15)	1.9 (0.042)	9.5 (0.24)	10.4 (0.0500)	22.3 (0.534)	2.6 (0.016)	34.9 (0.800)	8.3 (0.13)	8.2 (0.047)	9.9 (0.18)
	Partial crops	Native	11.2	5.3	8.9	7.5	1.1	5.6	5.1	1.6	6.1	6.1	15.8	1.5	17.9	5.5	5.7	6.1
		+	7.0	8.5	13.4	10.5	1.6	8.1	8.0	1.4	9.7	11.1	23.1	1.7	26.0	8.4	8.2	7.6
		–	3.2	8.3	12.6	8.6	1.6	7.8	7.4	1.0	9.3	10.4	21.7	1.7	23.4	8.1	7.8	7.0
	Whole crops	Native	10.5 (0.564)	5.3 (0.17)	8.8 (0.37)	7.3 (0.39)	1.1 (0.0097)	5.6 (0.23)	4.9 (0.13)	1.5 (0.054)	5.9 (0.12)	5.8 (0.26)	14.8 (0.0640)	1.4 (0.038)	17.7 (1.01)	5.4 (0.11)	5.4 (0.14)	5.8 (0.31)
		+	4.2	6.1	9.4	7.5	1.2	5.7	5.7	1.1	7.0	8.1	16.2	1.1	15.8	6.2	6.0	5.5
		–	3.0	6.1	9.3	6.5	1.2	5.6	5.5	0.6	6.9	7.8	16.0	1.2	15.8	6.1	6.0	5.7
78	Seeds	Native	17.4 (0.516)	8.8 (0.23)	15.0 (0.493)	14.7 (0.745)	1.8 (0.087)	9.9 (0.40)	7.7 (0.31)	2.0 (0.060)	9.5 (0.26)	9.6 (0.33)	22.9 (1.48)	2.8 (0.21)	35.0 (1.28)	8.6 (0.35)	8.4 (0.29)	10.2 (0.449)
		+	13.4 (0.393)	9.2 (0.24)	15.2 (0.474)	14.8 (0.479)	1.7 (0.073)	10.0 (0.275)	7.7 (0.31)	1.1 (0.022)	9.9 (0.30)	10.8 (0.360)	23.5 (0.694)	2.7 (0.13)	33.1 (1.04)	8.8 (0.28)	8.7 (0.41)	8.8 (0.35)
		–	10.4	9.9	16.2	15.2	1.8	10.8	8.0	2.0	10.6	12.1	24.2	2.8	32.1	9.5	9.2	9.3
	Partial crops	Native	11.8 (0.168)	6.1 (0.11)	10.4 (0.105)	8.8 (0.12)	1.3 (0.026)	6.7 (0.047)	5.5 (0.14)	1.7 (0.086)	6.8 (0.039)	6.6 (0.081)	16.5 (0.131)	1.7 (0.054)	21.3 (0.566)	6.4 (0.057)	6.2 (0.054)	6.9 (0.054)
		+	3.6	6.8	10.8	8.9	1.3	6.7	6.0	1.5	7.6	7.7	16.8	1.4	17.8	6.9	6.7	6.4
		–	3.2	7.0	10.9	9.1	1.3	6.6	6.1	0.9	7.7	8.2	16.8	1.6	20.1	7.2	6.1	4.8

	Whole crops	Native	7.2 (2.4)	4.5 (1.2)	7.6 (2.0)	6.4 (1.9)	0.9 (0.3)	4.8 (1.4)	4.1 (0.81)	1.3 (0.14)	5.0 (1.2)	4.9 (0.98)	11.6 (3.01)	1.3 (0.32)	14.9 (4.97)	4.8 (0.93)	4.5 (0.96)	5.1 (1.3)
		+	2.4	5.3	8.2	7.3	1.0	5.0	4.8	1.1	5.9	6.3	13.1	1.2	15.2	5.7	5.0	4.0
		–	1.9	5.1	7.7	6.4	1.0	4.7	4.4	0.6	5.7	6.0	12.2	1.2	14.5	5.4	4.6	3.8
79	Seeds	Native	18.1	9.0	15.5	15.3	1.9	10.2	8.0	2.1	9.8	9.5	24.4	3.0	35.7	9.2	8.8	10.5
		+	16.4	9.7	16.1	15.8	1.9	10.7	8.4	1.8	10.5	10.7	25.8	3.0	37.1	9.9	9.2	10.2
		–	14.0	9.7	16.2	15.3	1.9	10.8	8.2	2.0	10.4	11.6	24.4	2.9	37.2	9.9	9.0	10.3
	Partial crops	Native	10.3	5.9	9.6	9.0	1.2	6.3	5.2	1.6	6.5	6.1	15.5	1.9	20.5	6.1	5.4	6.6
		+	7.1	7.0	11.1	10.7	1.3	7.3	5.9	1.5	7.6	7.3	17.9	1.8	23.5	7.0	6.8	6.1
		–	6.8	7.5	12.3	11.9	1.4	8.0	6.4	1.7	8.4	8.1	19.6	1.9	20.8	7.7	7.2	7.2
	Whole crops	Native	9.7	5.5	9.2	8.7	1.2	6.1	5.1	1.5	6.1	5.9	14.8	1.7	20.1	5.9	5.3	6.4
		+	2.2	4.2	6.4	6.7	0.9	3.9	4.1	1.0	4.9	5.1	11.1	1.0	13.0	4.9	4.3	3.2
		–	5.5	5.7	9.3	8.3	1.1	6.0	4.9	1.1	6.3	6.2	14.9	1.4	19.3	5.8	5.4	6.0
86	Seeds	Native	17.8 (0.0311)	8.6 (0.0041)	14.2 (0.105)	14.3 (0.128)	1.7 (0.028)	9.5 (0.058)	7.3 (0.048)	1.9 (0.019)	9.3 (0.017)	8.6 (0.061)	23.1 (0.264)	2.8 (0.068)	33.4 (0.325)	8.7 (0.060)	7.8 (0.021)	9.4 (0.14)
		+	19.3	9.3	15.4	15.7	1.9	10.3	8.0	2.0	10.1	9.3	25.1	3.0	36.2	9.4	8.8	10.2
		–	17.7	9.0	14.8	12.2	1.8	9.9	7.6	1.9	9.7	9.2	23.9	2.4	33.7	9.1	8.6	9.8
	Partial crops	+	13.1	8.3	13.5	11.9	1.6	8.9	6.9	1.7	9.0	8.7	22.1	2.1	29.8	8.3	7.8	8.5
		–	8.9	6.6	10.8	9.1	1.2	7.1	5.5	1.4	7.3	6.9	17.5	1.7	23.1	6.6	6.4	7.1

n.a. = not analyzed (sample material was not available for this analysis). Amino acid concentrations are given in g/kg dry matter. Maturity stages are encoded according to Meier (45). Analyses were performed using one sample per variant, unless otherwise indicated. Means of two biological replicates per variant are highlighted grey; means of three replicates per variant are highlighted blue. The standard deviation of means is given in brackets.

Table S10. Ensiling characteristics and concentrations of ammonia, ammonia nitrogen (NH_3-N), lactic acid, acetic acid, and alcohols of silages of pea seeds, partial crop peas, and whole crop peas, harvested at five maturity stages, with or without addition of bacterial inoculant.

Maturity (BBCH)	Crop variant	Inoculant	pH	pH_{AS}	ASTA	Ammonia	NH_3-N	Lactic acid	Acetic acid	Methanol	Propanol	Ethanol	Butanol
76	Seeds	+	4.2 (0.043)	n.a.	n.a.	3.5	7.3	85.0	4.9	0.6	0.7	2.8	8.7
		–	5.7 (0.23)	n.a.	n.a.	6.1	11.8	2.7	8.9	0.7	0.8	3.3	21.9
	Partial crops	+	4.1 (0.0071)	8.3 (0.078)	30 (2.9)	2.9	8.2	143.8	8.1	0.7	0.9	4.3	11.3
		–	4.1 (0.0071)	7.4 (0.42)	104 (1.07)	4.0	11.6	153.6	14.3	0.7	0.7	4.2	5.0
	Whole crops	+	4.1 (0)	8.6 (0)	25 (6.1)	2.3	7.8	129.8	5.5	0.7	0.7	4.5	2.2
		–	4.2 (0.029)	6.6 (1.8)	123 (9.90)	4.1	14.0	140.3	18.1	0.7	0.8	4.7	3.6
77	Partial crops	+	4.2 (0)	7.0 (2.2)	112 (29.4)	2.9	7.5	123.2	11.6	0.6	0.6	3.7	6.4
		–	4.3 (0.015)	4.5 (0.015)	144 (8.49)	2.0	5.6	112.7	13.0	0.7	0.7	3.7	10.8
	Whole crops	+	4.3 (0.015)	7.5 (1.5)	111 (19.5)	3.2	10.9	112.7	12.1	0.7	0.6	4.0	4.2
		–	4.3 (0.022)	7.1 (0.036)	126 (1.07)	2.3	8.0	115.1	15.8	0.6	0.5	3.7	4.6
78	Seeds	+	4.3 (0.015)	4.4	152	1.0 (0.2)	2.3 (0.40)	53.5 (7.10)	3.7 (0.54)	0.4 (0.02)	0.6 (0.2)	2.0 (0.034)	7.5 (1.7)
		–	4.6 (0.18)	4.9	168	2.4	5.4	43.4	7.2	0.4	0.5	2.1	14.4
	Partial crops	+	4.4 (0.036)	6.2 (2.4)	156 (17.4)	2.5	8.0	50.0	16.7	0.5	0.5	3.2	3.6
		–	4.4 (0.0071)	4.9 (0.64)	138 (42.5)	2.0	6.3	43.2	11.7	0.6	0.7	3.0	6.8
	Whole crops	+	4.4 (0.0071)	6.4 (2.8)	146 (31.5)	1.8	6.4	72.3	13.6	n.d.	n.d.	2.4	3.2
		–	4.5 (0.0071)	4.5 (0.050)	168 (0)	2.1	8.2	89.1	14.3	n.d.	n.d.	2.9	4.2
79	Seeds	+	4.3	n.a.	n.a.	0.7	1.4	35.7	4.1	0.5	0.2	1.5	9.6
		–	4.6	n.a.	n.a.	1.0	2.2	22.9	3.5	0.5	0.2	1.5	18.2

	Partial crops	+	4.5 (0.0071)	4.5 (0.022)	168 (0)	1.3	3.4	43.2	9.8	0.4	0.5	1.9	3.3
		–	4.7 (0.015)	4.8 (0.0071)	168 (0)	1.8	4.8	31.8	16.8	0.4	0.4	2.0	6.0
	Whole crops	+	4.6 (0.029)	4.6 (0.043)	168 (0)	1.4	4.2	30.5	16.5	0.3	0.4	2.2	3.4
		–	4.7 (0)	4.8 (0)	168 (0)	1.4	4.4	66.2	15.1	0.4	0.6	2.3	3.9
86	Seeds	+	5.9 (0.14)	6.8 (0.021)	67 (2.1)	n.d.	n.d.	n.d.	0.6	0.3	0.1	1.0	2.0
		–	6.1 (0.012)	6.7 (0.12)	87 (4.1)	0.1	0.1	2.2	0.5	0.3	0.1	1.0	1.3
	Partial crops	+	4.7 (0.0071)	4.8 (0.0071)	168 (0)	0.7	2.0	45.2	8.9	0.4	0.1	1.6	4.7
		–	5.0 (0.064)	6.2 (1.5)	168 (0)	0.8	2.5	16.5	4.9	0.4	0.2	1.8	3.4

AS = aerobic storage (i.e., silages were stored 7 d under aerobic conditions), ASTA = aerobic stability, n.a. = not analyzed (sample material was not available for these analyses), n.d. = not detected, pH_{AS} = pH measured immediately after 7 d of aerobic storage.

Concentrations of ammonia, lactic acid, acetic acid, and volatile alcohols are given in g/kg dry matter, NH_3-N is given as % of total nitrogen, ASTA is given in h until the temperature difference between material and environment exceeds 3 K. Maturity stages are specified in Table 1 and encoded according to Meier (45). Analyses were performed using one sample per variant, unless otherwise indicated. Means of two biological replicates per variant are highlighted grey; means of three replicates per variant are highlighted blue; the standard deviation of means is given in brackets.
